# Positive Outcomes of a Comprehensive Health Literacy Communication Training for Health Professionals in Three European Countries: A Multi-centre Pre-post Intervention Study

**DOI:** 10.3390/ijerph16203923

**Published:** 2019-10-15

**Authors:** Marise S. Kaper, Andrea F. de Winter, Roberta Bevilacqua, Cinzia Giammarchi, Anne McCusker, Jane Sixsmith, Jaap A.R. Koot, Sijmen A. Reijneveld

**Affiliations:** 1 Department of Health Sciences, University of Groningen, University Medical Center Groningen, 9700 RB Groningen, The Netherlands; 2 IRCCS INRCA (The National Institute of Health and Science on Aging), 60124 Ancona, Italy; 3 The Regional Agency for Health (ARS of the Marche Region), 60125 Ancona, Italy; 4 Belfast Healthy Cities, Belfast BT1 1RD, UK; 5 Health Promotion Research Centre, National University of Ireland Galway, Galway H91 TK33, Ireland

**Keywords:** health literacy, person-centred communication, health promotion, health professional, capacity building, continuing professional education, empowerment, shared decision-making, self-management

## Abstract

Many professionals have limited knowledge of how to address health literacy; they need a wider range of health literacy competencies to enhance empowerment and person-centred prevention. We evaluated whether: (1) a comprehensive health literacy training increased self-rated competencies of health professionals to address health literacy related problems and support the development of people’s autonomy and self-management abilities after training and 6–12 weeks later, (2) professionals were satisfied with the training, (3) outcomes differed for the three participating European countries. Health professionals (N = 106) participated in a multicentre pre-post intervention study in Italy, the Netherlands and Northern Ireland. The 8-hour training-intervention involved health literacy knowledge, the practice of comprehensible communication skills, shared decision-making, and enhancing self-management. Self-rated health literacy competencies and training satisfaction were assessed at baseline, immediately after training and 6-12 weeks later, and analysed by multi-level analysis. Professionals’ self-rated health literacy competencies significantly improved following training in all three countries; this increase persisted at 6-12 weeks follow-up. The strongest increase regarded professional’s skills to enhance shared-decision making and enabling self-management after training and follow-up respectively. Professionals perceived the training as relevant for practice. Competency increases seemed to be consistent across countries. In three countries, professionals’ self-rated health literacy competencies increased following this comprehensive training. These promising findings should be confirmed in a further full effect study. Implementation of this training in European education and health care may improve person-centred communication by professionals and might help to tackle health literacy related problems and to strengthen people’s abilities in achieving better health outcomes.

## 1. Introduction

By communicating more effectively, health care professionals have the possibility to improve the quality of person-centred prevention and health care for people with limited health literacy. To date however, many health professionals lack sufficient competencies to address health literacy related problems of their patients and to strengthen their empowerment [1,2,3]. People with limited health literacy, especially those who are older and have a lower social economic status [4,5,6], encounter problems with understanding information, communicating with health professionals, and engaging in self-management activities [6,7,8]. Health literacy has been defined as “the degree to which people are able to access, understand, appraise and communicate information to engage with the demands of different health contexts in order to promote and maintain good health across the life-course” [9]. Nearly 50% of people in the general population surveyed across eight European countries were found to have problematic or inadequate health literacy [5]. Limited health literacy skills of people pose a challenge to the provision of high quality health care and are consistently associated with poorer health outcomes [6], higher mortality rates [10], and lower quality of life in people with long-term chronical illnesses [11]. In effective conversations, health professionals can enhance people’s health literacy skills, thereby contributing to increased adherence, patient safety, and improved quality of life and health outcomes [12,13,14]. 

When health professionals have better competencies to address health literacy related problems in conversations with people, this can contribute to the effectiveness of prevention and to the management of the increasing demands on health care [15,16]. Many countries have an ageing population, living with a higher prevalence of chronic illness [17]. People place a greater demand on health care services, and health care systems need to support ageing people to stay active and enhance their autonomy and in managing their health in their daily lives [18,19]. As a consequence, health professionals and social workers need a wider range of competencies to identify health literacy problems and communication skills to enhance patients’ understanding, autonomy and self-management [20]. Currently, many professionals have but limited knowledge of health literacy related problems and the communication skills to address it [1,2].

Several reviews [21,22] have reported that health literacy training for professionals increases their knowledge and awareness of health literacy related problems and the use of clear communication strategies to enhance patient understanding of information (e.g., functional health literacy) [23,24]. Nevertheless, the available training-interventions rarely addresses the broader competencies of professionals which are needed to strengthen the autonomy and self-management of people with limited skills [3,23,24] relating to interactive health literacy (i.e., ability to communicate and apply health information) and critical health literacy (i.e., information analysis and controlling one’s health). 

Given the current lack of professional training programs to strengthen the empowerment and self-management of their patients, a comprehensive program was developed to train health professionals to address health literacy related problems and enhance the functional, interactive and critical health literacy skills of their patients [25]. This training was positively evaluated among a small sample of health professionals from three European countries working in clinical practice. In this study, we have extended this comprehensive training program [25] to professionals working in clinical and community settings, and have evaluated its training outcomes in a larger sample of health professionals. Our objectives were to evaluate whether: (1) a comprehensive health literacy training increased self-rated competencies of health professionals to address health literacy related problems and support the development of people’s autonomy and self-management abilities after training and 6-12 weeks later, (2) professionals were satisfied with the training, (3) outcomes differed for the three participating European countries.

## 2. Materials and Methods

### 2.1. Design

We conducted a multicentre pre-post intervention study in three European countries, i.e., Italy, The Netherlands and Northern Ireland, to evaluate self-rated outcomes of 8 hours of health literacy communication training among a multi-disciplinary sample of health professionals working in clinical and community settings. The study was conducted in the period from February 2017 to October 2017.

### 2.2. Setting and Participants

We used convenience sampling to recruit qualified health professionals from various backgrounds working in clinical- and community settings, such as hospitals and dialyze-centres, ambulatory community treatment centres, community networks, rehabilitation services, and home care. The reason for our sampling approach was that health professionals, who often work together in teams, expressed that all their colleagues would need to apply health literacy skills in their daily conversations with patients, in order to reduce communication problems [5]. The researchers who conducted the study in each country, approached health professionals from partner health care organizations. Professionals were contacted via newsletters, professional networks, email and phone. We recruited professionals with various backgrounds such as nursing, physiotherapy, psychology, medicine, health promotion and social work. The inclusion criteria for health professionals was that they had to have frequent conversations with patients or clients from groups being at risk for low health literacy (such as elderly or low SES) with the aim to promote health or to provide health care or treatment. Exclusion criteria were professionals not having frequent conversations with patients or clients, not working in the health care sector, and being a student. 

Professionals received a written invitation with information on the study and the health literacy communication training. They in return provided written informed consent for participation in the study. The study was conducted in accordance with the Declaration of Helsinki and appropriate ethical guidelines in each country. The study-protocol was approved by the Ethics Committees of the University of Ulster, Filter Committee Nursing and Health Research (dated 7-1-2017) in Northern-Ireland, and exempted from further independent ethical review by the University Medical Center Groningen, Medical Ethical Committee (registration number 2016/909) in the Netherlands and Italy.

The power analysis showed that at least 93 participants (N = 31 per country) were needed to detect a significant difference of 1 point change between the pre- and post-measurement of perceived health literacy competencies overall and within each group, with an alpha of 0.05, a power of 0.80 and a standard deviation of 1.5 point. Taking into account a loss-to-follow up of 20%, we required 112 professionals.

### 2.3. Intervention 

The intervention involved health literacy communication training for professionals to enhance functional, interactive and critical health literacy of their patients by: (1) increasing evidence-informed knowledge of health literacy, (2) enhancing person-centred communication skills to address health literacy, and (3) sustaining the application of these skills in practice (Table 1). Facilitators experienced in health literacy communication training moderated the training sessions, and they were familiar with the country-specific context. The intervention involved five training sessions in the local language, with an additional 2 hours of self-study. In week one participants attended sessions 1 and 2, in week 2 sessions 3 and 4, and in week 6 session 5, allowing time in between to practice skills. 

To train the professionals we used various interactive learning approaches [21,22,26,27]. After an interactive introduction on health literacy, professionals participated in group discussions, which helped them to apply evidence-based health literacy knowledge to their practice experience. Practice-based role-played conversations involving patients with limited health literacy were used to strengthen professionals’ communication skills. We based the role-play scenarios on health literacy issues identified in the literature [6,28] and on practice examples identified through interviews with health professionals. Role-play scenarios were critically reviewed by the facilitators and the professionals who were interviewed. In the training, the professional and patient roles were played by one professional each, and observed by fellow professionals and facilitators. Patient roles described what patients with limited health literacy experience and contained instructions to display signs of limited health literacy: consult the professional with already aggravated problems, be unable to judge information or understand instructions, be silent, and ask few questions. Role-play instructions also contained clinical information. In one role-play scenario for example, professionals had a conversation with a low health literate patient having diabetes mellitus type 2. Professionals explained to this patient why adapting to a healthier diet is important and discussed step by step how this patient could change his behaviour. In the training, professionals also had the opportunity to role-play and reflect on a complex health literacy situation they had experienced in their own practice. In between sessions, we also encouraged professionals to record and reflect on a conversation with one of their patients or to be observed by a colleague, as far as patients provided permission to do so. In the follow-up session, professionals put forward an action plan to apply communication skills in daily practice. 

### 2.4. Procedure

Professionals completed three self-report questionnaires on health literacy competencies: at baseline, at first follow-up immediately after the training, and second follow-up six to twelve weeks post- training (See Supplementary Materials for the three questionnaires). The purpose of the measurement after six weeks was to measure professionals’ competency levels, after a period of sufficient length to let the professionals apply the learned communication strategies in conversations with their patients. We measured self-rated health literacy knowledge, attitude, confidence and application of communication skills at these three time points. Satisfaction with the training was measured at first follow-up. 

### 2.5. Background Information

At baseline, we measured background characteristics such as gender, age, professional background and years of working experience, and educational level. Furthermore, we asked professionals how often they worked with patients with limited health literacy, and how often they had previously attended health literacy and communication training. Questions were rated on a 5-point scale (1 = “never” to 5 = “very often”). 

### 2.6. Outcomes

The primary outcomes were the perceived competencies in communication with patients with limited health literacy; these competencies were health literacy knowledge, attitude, confidence, and health literacy communication skills. We measured health literacy competencies with a self-rated questionnaire (See Supplementary Materials for the three questionnaires). We selected the relevant subscales from questionnaires which were used in previous studies on health literacy training interventions for (future) professionals, or derived from frameworks of communication strategies, as far as no suitable questionnaires were available which matched with the contents of our training [29,30,31,32,33,34]. 

The questionnaire encompassed four subscales, which corresponded with the training-outcomes related to the health literacy competencies:

(1) The subscale “Health literacy knowledge [29,33,34]” involved six questions [29] on a 7-point scale (1 = “strongly disagree” to 7 = “strongly agree”).
(2) The subscale “Attitude” towards health literacy involved four questions on a 7-point scale (1 = “strongly disagree” to 7 = “strongly agree”), which was derived from the Health Literacy Strategies Behavioural Intention Questionnaire (HLSBI) [1,35]. This questionnaire is based on the Theory of Planned behaviour [36,37]. The Cronbach’s alpha reported was 0.76, indicating adequate internal reliability and a number of relationships between variables were being explored [1,35].
(3) The subscale “Confidence” to use health literacy communication skills involved 11 questions [38] on a 5-point scale (1 = “not at all confident” to 5 = “very confident”).
(4) The subscale “Health literacy communication skills [15,39]” involved four categories with 16 questions in total on a 7-point scale (1= “never” to 7 = “every time”): the category “gathering information” had four questions [25], the category “providing information” had five items [29,33,34], the category “shared decision-making” [32] had four questions and the category “enabling self-management” had three items [39].

We used the subscales health literacy knowledge and skills related to providing information as the basis for our questionnaires, as they matched with the contents of the training. These questions all stem from the same questionnaire, which was applied with positive results in three previous studies on health literacy training. The subscales attitude and confidence were added from two other previous studies, as our basic questionnaire did not contain these scales. The categories with the questions for gathering information, shared-decision-making and enabling self-management were based on frameworks of communication strategies. Cronbach’s alphas for the four subscales ranged from 0.73 to 0.94, indicating good internal reliability.

The secondary outcome was professionals’ satisfaction with the training, which we evaluated at first follow-up. Professionals filled in seven evaluative questions [30] on a 7-point scale (1 = “strongly disagree” to 7 = “strongly agree”) and rated the trainers and the training on a scale from 1–10. In addition, professionals answered questions on the balance between theory and practice, and on the trainer, training level, group size and length of the training. 

### 2.7. Analysis 

Mean scores were imputed for nine participants who had missing values (maximum 50% of items) on a subscale of the primary outcomes. We assessed the background of participants from the three countries as well as differences between countries, using Chi-square tests, and F-tests in ANOVA. For each of the three measurements, we then calculated the mean scores of each subscale (by counting the total scores divided by the number of questions in each subscale). We then assessed changes in health literacy competencies between baseline and first follow-up and between baseline and second follow-up with regard to the primary outcome variables, using multi-level analyses. We measured professionals’ self-rated satisfaction with the training and assessed differences between countries using F-tests in ANOVA. We assessed whether changes of competencies between baseline and first follow-up and between baseline and second follow-up were modified by country and background characteristics (in the analysis we included the main effect of time, country and background characteristics and the interactions of time with country, and time with background characteristics).

## 3. Results

### 3.1. Background Characteristics

Health professionals from various disciplines such as medicine, nursing, physiotherapy, psychology, health promotion and social work, participated in the study. One hundred and six health professionals completed the questionnaire at baseline. Of these professionals, 93% filled in the questionnaire at first follow-up and 64% filled in the questionnaire at second follow-up. The health professionals in the three countries differed significantly regarding their background characteristics: age, gender, education level, and years in current position, and experience with patients with limited health literacy, and previous health literacy training (Table 2). 

### 3.2. Positive Outcomes Following Health Literacy Communication Training

Overall, after the training (baseline – first follow-up) we found a significant improvement in professionals’ self-rated health literacy competencies: health literacy knowledge, attitude, confidence and health literacy communication skills (Table 3). 

The greatest changes were found in the communication skills shared decision-making and enabling self-management. The improved level of health literacy competencies persisted 6-12 weeks after the training on all aspects (baseline – second follow-up). The greatest improvements were found in the self-rated skills providing information, shared decision-making and self-management. 

### 3.3. Secondary Outcomes Regarding Training Satisfaction

Overall, professionals positively evaluated the health literacy communication training (Table 4). Positive evaluations were rated on the same level in all countries, indicated by the non-significant p-values. Dutch professionals provided positive, but somewhat lower ratings for the trainers and the overall training, compared to Italian and Northern Irish professionals. 

In addition, the majority of professionals across three countries indicated that the balance between theory and practice was good, that the trainer connected well to the practice situation of the professionals and challenged them to participate actively. Professionals indicated that the training level, group size and length were good. They considered the training objectives to have been reached, and concluded that they could use the training in their daily practice.

### 3.4. Consistent Training Outcomes in Three Countries

Overall, the increase in health literacy competencies seemed to be consistent among health professionals from the three countries. One’s country did not modify the change in health literacy competencies. The estimated main effects of country were not significant (ranges of B; 0.00 to 0.38, and *p*; 0.134 to 0.986), nor were the interactions of time by country (ranges of B; 0.01 to 0.39, and *p*; 0.09 to 0.98). Background characteristics did not modify the change in health literacy competencies over time.

## 4. Discussion

After attending this comprehensive health literacy training, we found a significant increase in self-rated competencies of health professionals to address health literacy related problems and skills to promote understanding of information, and support the development of autonomy and self-management abilities in conversations with patients. Increases in competencies and skills persisted to twelve weeks after the training. Health professionals gave positive evaluations of the training and considered it relevant for their practice. They suggested extending this training and providing it to students. In three countries, we found similar positive training outcomes among various health professionals working in clinical and community settings. 

The training increased professionals’ self-rated competencies to address limited health literacy and to support empowerment of their patients, with improvements persisting after six to twelve weeks follow-up. Our study builds upon the results found in previous studies and extends these findings. Results in our study showed similar improvements in health literacy knowledge and comprehensible communication, in comparison with previous studies [15,21,29]. Its comprehensive training components augment these positive outcomes among professionals by addressing the complete range of functional, interactive and critical health literacy [3,25], thus supporting a wider range of professional competencies to support the development of the autonomy and self-management abilities of patients [40,41]. This may be crucial in promoting the health and well-being of vulnerable home-dwelling groups [11,42,43].

Health professionals also gave positive evaluations of the training and considered it helpful and relevant for addressing health literacy in conversations with their patients. The positive evaluations and the perceived relevance of the training are important facilitators for its application [44,45]. Learning of professionals was facilitated in congruence with their (cultural) context. The training was, for example, provided in the local language, and as they were familiar with the local context, facilitators could relate feedback to the practice of professionals. In our view, the health literacy components and interactive learning approach contributed to the positive evaluations and the improved health literacy competencies. The interactive learning in multiple training sessions was a useful method to improve self-rated health literacy knowledge, attitudes and communication skills [22,46]. The same applies to the action plan that we used to embed health literacy competencies in their practice [21,46]. The training components and interactive learning approach may be an explanation for the positive and persistent training outcomes six to twelve weeks after the training. [34,47]. 

Health literacy competencies of professionals in all three countries increased to a similar degree, despite significant differences in the background characteristics of participants. This finding may support the transferability of the training to a wider range of European health professionals. Moreover, several other studies support our finding that despite differences in countries, professionals can hold relatively consistent views on problems, attitudes and practice situations [48,49,50]. In Europe, two frameworks have been developed to promote harmonization of training in patient-centred communication in the education of health professionals [51,52]. Our study reinforces the evidence on the value of such initiatives [22]. To the two European frameworks it adds competencies to address health literacy and support the development of autonomy and self-management skills of vulnerable populations [51,52]. 

### 4.1. Strengths and Limitations

This study has several strengths. We used a multi-centre pre-post design to evaluate outcomes of the training in three countries, and among professionals from various backgrounds. Moreover, a follow-up measurement 6–12 weeks after the training allowed us to estimate outcomes after professionals had put into practice their health literacy knowledge and skills. 

Our study also had some limitations; a first one is being the lack of a control group. The positive results in self-rated outcomes may thus stem from an effect of time, or be a learning effect from filling in questionnaires. A second limitation is that we did not conduct measurements after a follow-up of a half year to over one year, to see if the changes in health literacy competencies also persisted after six to twelve weeks. Several studies have shown interventions for health professionals to improve knowledge, attitudes, and skills up until a follow up period of a half year [22,53], yet another study found significant drops after one year or more [34]. Third, at the second follow-we had some dropouts, which may have caused some selection because of only the most motivated participants answering the evaluation questionnaire. Fourth, we fully relied on self-report and not, for example, on observation. This may have led to some overestimation of competencies, as several studies have shown that health professionals tend to overestimate the actual level of health literacy skills of patients [54,55], the level of their own skills and attitudes when compared to general observation [56], and during conversations patients and doctors can have different perceptions of the communication skills of doctors [57]. Fifth, effects of the training could vary among more specific and homogeneous samples. The size of our sample did not allow us to assess an effect modification among specific and more homogeneous target groups of health professionals, for example physicians and physician assistants.

### 4.2. Implications for Practice and Research 

This comprehensive training is relevant to a wide range of health professionals; it may help to promote the autonomy and self-management skills of people with limited health literacy and has the potential to strengthen the effectivity of person-centred prevention and treatment in clinical and community settings. This training can be easily adapted to different country-specific contexts and may enhance the quality of health literacy education for future health professionals. This evidently calls for wider transfer of this training to other European countries.

To enrich future health literacy trainings and facilitate exposure to potential health literacy issues, we suggest e-teaching tools with (standardized) videotaped scenarios based on health literacy issues identified in the literature and practice situations. After viewing the scenario, health professionals can practice communication skills by videotaping their own response to this scenario and receive feedback. We also encourage for example to establish panels of patients with low health literacy. As part of a training, these panel-members could share their experiences with health professionals or show to professionals what they encounter when viewing a website or visiting a hospital.

Future extensions of the training would allow addressing more advanced levels of health literacy competencies. This advanced level would relate to abilities such as diagnostics of health literacy problems in complex or ambiguous situations, sensitivity and flexibility in using various communication strategies, implementing health literacy practices in organisations, and coaching other employees.

The outcomes of this comprehensive health literacy training need further confirmation in a full effect study, with preferably a randomized controlled design, and with a still longer follow up period to assess if changes in health literacy competencies and practices persist over time. 

Future studies could analyse an effect modification related to the training outcomes among more specific and homogeneous target groups, such as physicians and physician assistants, and future health professionals in specific educational settings. This may highlight where specific adaptations are needed to further improve training-outcomes. Research should also determine the effects of health literacy training for professionals on patient outcomes, by examining their satisfaction with conversations, recall of information, the quality of health decisions patients were making concerning the management of their condition(s) and their adherence to agreed-upon treatment protocols.

## 5. Conclusions 

This study found that a comprehensive training increased self-rated health literacy competencies of health professionals, with improvements potentially persisting until six up to twelve weeks after the training, in particular regarding communication skills to support peoples’ autonomy and self-management abilities. These findings should be confirmed in a further full effect study, with preferably a randomized control design. Next, health professionals were positive about the training, and outcomes were consistent across three countries. This study indicates that a wider transfer of this health literacy training to European health care practice and education might offer a great opportunity to promote effective communication with people with limited health literacy.

## Figures and Tables

**Table 1:** Overview of the Health Literacy Communication Training program [25].

Session	Learning objectives	Contents
1. Evidence informed knowledge of health literacy (2 h).	A. To inform and educate: Professionals know about health literacy problems, and interventions to tackle health literacy problems.	Identify limited health literacy skills of patients: - Define functional, interactive and critical health literacy - Discuss the consequences, prevalence and impact of limited health literacy on patients. - Assess written education materials for limited health literacy - Describe cues and questions to identify health literacy
2. Gathering and providing information to address functional health literacy (1.5 h).	B. To teach skills: Professionals develop patient-centred communication skills to address problems with health literacy.	Demonstrate skills to gather and provide information: - Gather information by active listening, open questions, establish health literacy level, observe non-verbal signs, and explore emotions and potential feelings of shame. - Provide and prioritize information, use plain language, speak slowly, verify with teach back if patients understand information
3. Shared decision making to address interactive health literacy (1.5 h).	idem	Demonstrate shared decision-making skills: - Invite patients into shared decision-making and make them aware they have a choice. - Facilitate and educate patients to participate in decision making if needed; i.e. how to disclose their concerns, ask questions and state their preferences. - Explain options; provide comprehensible information relating to prior knowledge. Discuss harms and benefits. - Involve the patient in decision making; consider pros and cons and individual considerations.
4. Self-management to enhance critical health literacy (1.5 h).	idem	Demonstrate skills to enable self-management: - Discuss how to prepare for self-management. - Explore patient’s readiness for behaviour change and barriers to adherence. - Incorporate patient’s perspective to enable self-management; formulate personal goals and strengthen self-efficacy. - Provide realistic instructions tailored to prior knowledge of patients and their living situation. - Discuss required follow up: monitor self-care, review information and arrange of support.
5. Applying and sustaining health literacy communication (1.5 h).	C. To support behaviour change: Professionals adopt, change and maintain behaviour to address health literacy problems.	Demonstrate sustainable application of health literacy communication skills into practice: - Discuss and share experiences on application of health literacy communication. - Develop a practical tool or action plan to sustain communication in daily practice.

**Table 2:** Background characteristics of professionals by country at baseline.

Characteristics	Total	Italy	Netherlands	N. Ireland	*p*
Age^1^ (M; SD)	44.29 (11.86)	50.11 (9.78)	39.44 (11.94)	43.60 (11.32)	<0.001
Years in current position ^1^	12.15 (10.98)	21.64 (10.49)	4.93 (5.014)	7.25 (5.92)	<0.001
How often do you as professional ^2^: - work with patients limited health literacy^1^	3.68 (1.06)	3.75 (1.11)	4.05 (0.61)	3.13 (1.23)	0.001
- Previously health literacy training attended ^1^)	1.56 (0.81)	1.22 (0.49)	1.67 (0.90)	1.81 (0.87)	0.006
- Previously attended communication training ^1^	2.71 (0.95)	2.53 (0.97)	2.81 (0.95)	2.79 (1.06)	0.377
					
Gender (female) ^2^ (n; %)	97 (92%)	28 (78%)	39 (100%)	30 (97%)	0.001
Education level ^2^					<0.001
- High school or equivalent	3 (3%)	3 (8%)	-	-	
- Secondary vocational training	12 (11%)	-	12 (31%)	-	
- Bachelors (undergraduate)	39 (37%)	-	27 (69%)	12 (39%)	
- Masters (postgraduate)	39 (37%)	24 (67%)	-	15 (48%)	
- Other	13 (12%)	9 (25%)	-	4 (13%)	
Current profession ^3^					<0.001
- Nursing	52 (50%)	11 (31%)	39 (100%)	2 (7%)	
- Physiotherapy	9 (9%)	9 (25%)	-	-	
- Psychology	8 (8%)	7 (19%)	-	1 (3%)	
- Social work	19 (18%)		-	19 (63%)	
- Other health professions ^4^	17 (16%)	9 (25%)	-	8 (27%)	

^1^*p* = ANOVA F test; ^2^ Scale (1 = “never” to 5 = “very often”); ^3^ *p* = Chi square; ^4^ Other health professions include medical disciplines such as neurologist, physicians, general practitioners, and other professions involved in health promotion.

**Table 3:** Mean scores of self-rated health literacy competencies at baseline, first- and second follow-up, and estimates of the change between baseline and first-follow up, and baseline and second follow-up.

Health literacy competencies ^4^	Baseline	First follow-up		Second follow-up	
	M (SD) ^1^	M (SD)	B (95% CI) ^2^	M (SD)	B (95% CI) ^2^
Health literacy knowledge	4.97 (0.79)	5.85 (0.82)	0.87 (0.69; 1.05)	6.04 (0.53)	1.06 (0.86; 1.27)
Attitude towards health literacy ^3^	5.81 (0.86)	6.10 (0.84)	0.29 (0.15; 0.43)	-	-
Confidence HL	3.34 (0.73)	4.04 (0.53)	0.71 (0.56; 0.85)	4.19 (0.49)	0.84 (0.68; 1.01)
HL Communication skills:	4.35 (0.99)	5.21 (0.78)	0.87 (0.69; 1.05)	5.47 (0.87)	1.13 (0.93; 1.33)
- gathering information	5.08 (1.05)	5.68 (0.76)	0.63 (0.45; 0.82)	6.00 (0.79)	0.95 (0.73; 1.16)
- providing information	4.21 (0.97)	4.97 (0.77)	0.77 (0.58; 0.95)	5.39 (0.82)	1.19 (0.98; 1.40)
- shared decision making	4.08 (1.35)	5.19 (1.06)	1.12 (0.87; 1.37)	5.35 (1.26)	1.26 (0.98; 1.55)
- enabling self-management	4.07 (1.56)	5.07 (1.27)	1.00 (0.72; 1.29)	5.11 (1.23)	1.08 (0.75; 1.40)

^1^ M = Mean, SD = standard deviation; ^2^ B = parameter estimates of change between baseline and first-follow up, and baseline and second follow up. 95% CI = confidence interval. All parameter estimates p <0.001; ^3^ Attitude towards health literacy was only investigated at baseline and first follow-up; ^4^ Mean scores of the competencies, corresponding with the subscales, were calculated by counting the total scores divided by the number of questions in each subscale: (1) “Health literacy knowledge [29,33,34]” involved six questions on a 7-point scale (1 = “strongly disagree” to 7 = “strongly agree”). (2) “Attitude” [1] involved four questions on a 7-point scale (1 = ‘strongly disagree’ to 7 = ‘strongly agree’). (3) “Confidence” involved 11 questions [38] on a 5-point scale (1 = “not at all confident” to 5 = “very confident”). (4) The subscale “Health literacy communication skills [15,39]” involved 16 questions in total on a 7-point scale (1= “never” to 7 = “every time”): with the categories “gathering information” (four questions) [25], “providing information” (five questions) [29,33,34], “shared decision-making” (four questions) [32] and “enabling self-management” (three questions) [25,39].

**Table 4:** Mean scores of training satisfaction at first follow up.

Questions:	Total M (SD) ^1^	Italy M (SD)	Netherlands M (SD)	Northern-Ireland M (SD)	*p* ^2^
The training: - was appropriate for my educational level and experience.	5.65 (1.34)	5.56 (1.46)	5.63 (1.37)	5.79 (1.18)	0.784
- increased my knowledge about health literacy	6.02 (1.15)	5.85 (1.26)	5.83 (1.29)	6.45 (0.63)	0.056
- increased my confidence in communicating with patients with limited health literacy.	5.87 (1.08)	5.62 (1.30)	5.80 (1.05)	6.24 (0.69)	0.065
I found:				
- the role-play scenarios to be realistic.	5.54 (1.41)	5.44 (1.40)	5.40 (1.58)	5.83 (1.23)	0.431
- practicing with a standardized patient useful.	5.44 (1.35)	5.32 (1.30)	5.51 (1.54)	5.48 (1.18)	0.826
- the feedback following my role-play conversations useful.	5.79 (1.07)	5.64 (1.11)	5.86 (1.26)	5.90 (0.72)	0.580
- I will use the suggested communication strategies in my practice	5.98 (1.03)	6.00 (1.09)	5.74 (1.17)	6.24 (0.69)	0.155
- Mean rating trainers (Scale 1; 10)	8.80 (1.15)	9.63 (0.73)	7.75 (0.88)	9.06 (0.85)	0.000
- Mean rating training (Scale 1; 10)	8.42 (1.20)	9.37 (0.86)	7.71 (0.79)	8.21 (1.26)	0.000

^1^ M = Mean, SD = standard deviation; ^2^ *p* = ANOVA F test.

## Supplementary Materials

The following are available online at at https://www.mdpi.com/1660-4601/16/20/3923/s1, Questionnaires 1–3 on Health literacy communication training.
