# Supplementary material for: Positive Outcomes of a Comprehensive Health Literacy Communication Training for Health Professionals in Three European Countries: A Multi-centre Pre-post Intervention Study
Source: Int J Environ Res Public Health. 2019 Oct 15;16(20):3923. doi: 10.3390/ijerph16203923 (PMC6843857; doi:10.3390/ijerph16203923)
Supplement: Supplementary file 1 [file ijerph-16-03923-s001.pdf]

Questionnaires 1-3 on Health literacy communication training

Questionnaire 1.

Health Literacy Communication - before the training

Questionnaire 2

Health Literacy Communication

immediately following training

Questionnaire 3.

Health Literacy Communication six weeks following training

Questionnaire 1.

Health Literacy Communication - before the training

Fill in your unique participant number:	

Introduction

European research has found that nearly 50% of adults have limited health literacy. People with limited health literacy do not have the necessary skills to find, understand, or use information about health.

This training is designed to help health professionals to address problems with low health literacy in communication with patients. It is important for us to know how effective it is.

This questionnaire was therefore developed to study the quality of communication between health professionals and patients with limited health literacy. For the quality of the research we use different questionnaires. Because of this, some questions may look alike.

Please fill out this questionnaire three times: once before the start of the Health Literacy Communication Training, once immediately following the training, and once again six weeks after following the training. It takes about 15 minutes to answer the questions.

The questionnaire includes the following topics:

A. General questions about you and your organisation
B. Your knowledge of health literacy
C. Health literacy focused communication Skills
D. Attitude: Your opinion on using health literacy strategies
E. Your confidence in communication focused on health literacy

Questions

A. General questions about you and your organisation

1. What is your age in years?	……………. years
2. What is your gender?	o Male<br>o Female
3. What country do you live in?	o Italy<br>o Northern Ireland<br>o The Netherlands
4. What is the name of the organisation you work for?	

5. What is the highest level of completed education?	o Grammar school<br>o High school or equivalent<br>o Vocational/technical school (2 year)<br>o Some college<br>o Bachelor's undergraduate degree<br>o Master's postgraduate degree<br>o Professional degree<br>o Doctoral degree<br>o Other
6. What is your current position?	
7. How many years have you worked in this position?	………… years
8. How often do you work with patients with low health literacy?	o 1 – Never<br>o 2 – Rarely<br>o 3 – Occasionally<br>o 4 – Regularly<br>o 5 – Very often
9. Have you previously received education or training on health literacy?	o 1 – Never<br>o 2 – Rarely<br>o 3 – Occasionally<br>o 4 – Regularly<br>o 5 – Very often
10. Have you previously received education or training in communication?	o 1 – Never<br>o 2 – Rarely<br>o 3 – Occasionally<br>o 4 – Regularly<br>o 5 – Very often
Learning objectives in relation to communication about health literacy can be different for each person, for example "provide clearer written information", or "check frequently if patients understand information".<br><br>11. Can you indicate your learning objectives related to communicating with patients with low health literacy?	

B. Knowledge of health literacy

Please indicate how much you agree or disagree with the following questions:

Questions	1 Strongly disagree	2 Disagree	3 Some-what disagree	4 Neither agree nor dis-agree	5 Some -what agree	6 Agree	7 Strongly agree
12. I understand what it means for patients to have low health literacy.							
13. I know the prevalence of low health literacy.							
14. I know the groups that are more likely to be low health literate.							
15. I understand the health outcomes associated with low health literacy.							
16. I do a good job identifying low health literate patients.							
17. I am good at knowing whether or not my patients understand what I tell them.							

C. Health Literacy focused Communication skills

Indicate how frequently you use each technique when working with patients; on a scale from Never (1) to Every time (7).

**Gathering information**	**1 Never**	**2 Rarely**	**3 Occasio-nally**	**4 Some-times**	**5 Fre-quently**	**6 Usually**	**7 Every time**
18. Ask open questions							
19. Use active listening techniques to gather information							
20. Observe cues related to non-verbal communication							
21. Create a shame-free environment							
**Providing information**	**1 Never**	**2 Rarely**	**3 Occasio-nally**	**4 Some-times**	**5 Fre-quently**	**6 Usually**	**7 Every time**
22. Speaking slowly							
23. Using plain, non-medical language							
24. Show or draw pictures							
25. Limit the amount of information provided and repeat it							
26. Use the teach-back or show-me techniques *(the patients explains the information in his own words to check understanding).*							

**Shared decision making**	**1 Never**	**2 Rarely**	**3 Occasio-nally**	**4 Some-times**	**5 Fre-quently**	**6 Usually**	**7 Every time**
27. Convey awareness among patients that a choice exists in health care or treatment.							
28. Inform patients about health care or treatment options in more detail.							
29. Support patients to explore 'what matters most to them' after informing them on health care or treatment options.							
30. Train patients to participate in shared decision making.							
**Enabling self-management**	**1 Never**	**2 Rarely**	**3 Occasio-nally**	**4 Some-times**	**5 Fre-quently**	**6 Usually**	**7 Every time**
31. Assess barriers and facilitators relating to treatment compliance.							
32. Involve the patient in formulating personalized goals and action plans.							
33. Train patients to perform adequate self-management behaviour.							

D. Attitude: Your opinion on using health literacy strategies

Health literacy strategies are defined as the communication and teaching strategies that have been described as effective with low health literacy patients. These include, plain language communication, which is the avoidance of medical jargon, and Teach-Back, which is a teaching strategy that has the patient teach back to the provider the information just presented to them and also include strategies related to shared decision making and promoting self-management. Please read each question and circle the answer that best reflects your opinion on the use of health literacy strategies in clinical practice. Choose only one answer.

Questions	Scale					
34. My use of health literacy strategies with patients will result in patients having a better understanding of their illness and its treatment.	1 Likely	2	3	4	5	6	7 Unlikely
35. Improved patient understanding will improve patient outcomes.	1 Agree	2	3	4	5	6	7 Disagree
36. Use of health literacy strategies with patients would help patients stay healthy.	1 Agree	2	3	4	5	6	7 Disagree
37. My use of health literacy strategies with patients would be a…	1 Bad idea	2	3	4	5	6	7 Good idea

E. Your confidence in health literacy focused communication

With respect to the following questions, please indicate your level of confidence in using your health literacy communication skills

**How confident are you in your ability to:**	**1** **Not at all confident**	**2** **Slightly confident**	**3** **Neutral**	**4** **Moderately confident**	**5** **Very confident**
38. To communicate with patients who may have low health literacy					
39. Use instruments to identify patients with low health literacy					
40. Identify behaviours typically exhibited by people with low health literacy					
41. Judge appropriateness of written health information for patients with low health literacy					
42. Gather information from patients with low health literacy					
43. Provide clear information to patients with low health literacy					
44. Use the teach back or show me technique to check understanding of patients with low health literacy					
45. Create a shame free environment for patients with low health literacy					
46. Involve patients with low health literacy in shared decision making					

47. Train patients with low health literacy to participate in shared decision making					
48. Promote self-management skills in patients with low health literacy					

49. Do you have questions and/or comments?

Thank you very much for your cooperation!

Questionnaire 2

Health Literacy Communication

immediately following training

Fill in your unique participant number:	

Introduction

European research has found that nearly 50% of adults have limited health literacy. People with limited health literacy do not have the necessary skills to find, understand, or use information about health.

This training is designed to help health professionals to address problems with low health literacy in communication with patients. It is important for us to know how effective it is.

This questionnaire was therefore developed to study the quality of communication between health professionals and patients with limited health literacy. For the quality of the research we use different questionnaires. Because of this, some questions may look alike.

Please fill out this questionnaire two times: once immediately following the training, and once again six weeks after following the training. It takes about 15 minutes to answer the questions.

The questionnaire includes the following topics:

A. Your knowledge of health literacy
B. Health literacy focused communication Skills
C. Attitude: Your opinion on using health literacy strategies
D. Your confidence in communication focused on health literacy
E. Your evaluation of this training

Questions

A. Your knowledge of health literacy

Please indicate how much you agree or disagree with the following questions

**Perceived knowledge**	**1**<br>**Strongly disagree**	**2**<br>**Disagree**	**3**<br>**Some-what disagree**	**4**<br>**Neither agree nor dis-agree**	**5**<br>**Some -what agree**	**6**<br>**Agree**	**7**<br>**Strongly agree**
1. I understand what it means for patients to have low health literacy							
2. I know the prevalence of low health literacy							
3. I know the groups that are more likely to be low health literate							
4. I understand the health outcomes associated with low health literacy							
5. I do a good job identifying low health literate patients.							
6. I am good at knowing whether or not my patients understand what I tell them.							

B. Health Literacy focused Communication skills

Indicate how frequently you use each technique when working with patients, from Never (1) to Every time (7).

**Gathering information**	**1 Never**	**2 Rarely**	**3 Occasio-nally**	**4 Some-times**	**5 Fre-quently**	**6 Usually**	**7 Every time**
7. ask open questions							
8. use active listening techniques to gather information?							
9. Observe cues related to non-verbal communication							
10. Create a shame-free environment							
**Providing information**	**1 Never**	**2 Rarely**	**3 Occasio-nally**	**4 Some-times**	**5 Fre-quently**	**6 Usually**	**7 Every time**
11. Speaking slowly							
12. Using plain, non-medical language							
13. Show or draw pictures							
14. Limit the amount of information provided and repeat it							
15. Use the teach-back or show-me techniques *(the patients explains the information in his own words to check understanding).*							
**Shared decision making**	**1 Never**	**2 Rarely**	**3 Occasio-nally**	**4 Some-times**	**5 Fre-quently**	**6 Usually**	**7 Every time**
16. Make patients aware that they have a choice in health care or treatment.							
17. Inform patients about health care or treatment options in more detail.							

18. Support patients to explore 'what matters most to them' after informing on health care or treatment options.							
19. Train patients to participate in shared decision making.							
**Enabling self-management**	**1 Never**	**2 Rarely**	**3 Occasio-nally**	**4 Some-times**	**5 Fre-quently**	**6 Usually**	**7 Every time**
20. Assess barriers and facilitators relating to treatment compliance.							
21. Involve the patient in formulating personalized personalised goals and action plans.							
22. Train patients to perform adequate self-management behaviour.							

C. Attitude: Your opinion on using health literacy strategies

Health literacy strategies are defined as the communication and teaching strategies that have been described as effective with low health literacy patients. These include, plain language communication, which is the avoidance of medical jargon, and Teach-Back, which is a teaching strategy that has the patient teach back to the provider the information just presented to them and also include strategies related to shared decision making and promoting self-management. Please read each question and circle the answer that best reflects your opinion on the use of health literacy strategies in clinical practice. Choose only one answer.

Questions	**Scale**						
23. My use of health literacy strategies with patients will result in patients having a better understanding of their illness and its treatment.	1<br>Likely	2	3	4	5	6	7<br>Unlikely
24. Improved patient understanding will improve patient outcomes.	1<br>Agree	2	3	4	5	6	7<br>Disagree
25. Use of health literacy strategies with patients would help patients stay healthy.	1<br>Agree	2	3	4	5	6	7<br>Disagree
26. My use of health literacy strategies with patients would be a…	1<br>Bad idea	2	3	4	5	6	7<br>Good idea

D. Your confidence in health literacy focused communication

With respect to the following questions, please indicate your level of confidence in using your health literacy communication skills

**How confident are you in your ability to:**	**1**<br>**Not at all confident**	**2**<br>**Slightly confident**	**3**<br>**Neutral**	**4**<br>**Moderately confident**	**5**<br>**Very confident**
27. To communicate with patients who may have low health literacy					
28. Use instruments to identify patients with low health literacy					
29. Identify behaviours typically exhibited by people with low health literacy					
30. Judge appropriateness of written health information for patients with low health literacy					
31. Gather information from patients with low health literacy					
32. Provide clear information to patients with low health literacy					
33. Use the teach back or show me technique to check understanding of patients with low health literacy					
34. Create a shame free environment for patients with low health literacy					

35. Involve patients with low health literacy in shared decision making					
36. Train patients with low health literacy to participate in shared decision making					
37. Promote self-management skills in patients with low health literacy					

E. Your evaluation of this training.

For each question indicate which answer applies best to you by ticking the appropriate boxes.

38. Did you have a clear picture of the topics and objectives before the start of this training?	Yes	Partially	No
39. What was the balance between theory and practice (exercises, roleplays, assignments)?	Too much theory	Good	Too much practice
40. Were you sufficiently challenged by the trainer to participate actively?	Yes	Sometimes	No
41. Did the facilitator sufficiently relate back to your practice experience?	Yes	Sometimes	No
42. How difficult were the meetings?	Difficult	Good	Easy
43. Do you think you can use the contents of this training in your work?	Yes	Partially	No
44. How do you rate the length of this training?	Too long	Good	Too short
45. How do you rate the group size of this training?	Too large	Good	Too small
46. Did your supervisor pay attention to this training?	Yes	A little	No
47. Have the objectives of this training been achieved according to you?	Yes	Partially	No

48. Would you recommend this training to your colleagues?	Yes	Partially	No

Please indicate how much you agree or disagree with the following questions

**Questions**	**1 Strongly disagree**	**2 Dis-agree**	**3 Some-what disagree**	**4 Neither agree nor disagree**	**5 Some-what agree**	**6 Agree**	**7 Strongly agree**
49. The training was appropriate for my educational level and working experience.							
50. The training increased my knowledge about health literacy							
51. The training increased my comfort in communicating with patients with low health literacy.							
52. I found the roleplay descriptions to be realistic.							
53. I found practicing with a standardized patient useful.							
54. I found the feedback following my roleplay conversations useful.							
55. I will use the suggested communication strategies in my practice							

56. What did you learn in this training related to health literacy focused communication?
57. With which number from 1-10 would you rate the trainers?

Trainer 1<br>Trainer 2
58. With which number from 1-10 would you rate the training in total?
Do you have any advice or suggestions related to this training?

Thank you very much for your cooperation!

Questionnaire 3.

Health Literacy Communication six weeks following training

Fill in your unique participant number:	

Introduction

European research has found that nearly 50% of adults have limited health literacy. People with limited health literacy do not have the necessary skills to find, understand, or use information about health.

This questionnaire was therefore developed to study the quality of communication between health professionals and patients with limited health literacy after the training for health professionals. For the quality of the research we use different questionnaires. Because of this, some questions may look alike.

Please fill out this questionnaire once again six weeks after following the training. It takes about 15 minutes to answer the questions.

The questionnaire includes the following topics:

A. Your knowledge of health literacy
B. Health literacy focused communication Skills
C. Your confidence in communication focused on health literacy

Questions

A. Your knowledge of health literacy

Please indicate how much you agree or disagree with the following questions:

	1<br>Strongly disagree	2<br>Dis-agree	3<br>Some-what disagree	4<br>Neither agree nor disagree	5<br>Some -what agree	6<br>Agree	7<br>Strongly agree
1. I understand what it means for patients to have low health literacy							
2. I know the prevalence of low health literacy							
3. I know the groups that are more likely to be low health literate							
4. I understand the health outcomes associated with low health literacy							
5. I do a good job identifying low health literate patients.							
6. I am good at knowing whether or not my patients understand what I tell them.							

B. Health Literacy focused Communication skills

Indicate how frequently you use each technique when working with patients; from Never (1) to Every time (7);

**Gathering information**	**1 Never**	**2 Rarely**	**3 Occasio-nally**	**4 Some-times**	**5 Fre-quently**	**6 Usually**	**7 Every time**
7. ask open questions							
8. use active listening techniques to gather information							
9. Observe cues related to non-verbal communication							
10. Create a shame-free environment							
**Providing information**	**1 Never**	**2 Rarely**	**3 Occasio-nally**	**4 Some-times**	**5 Fre-quently**	**6 Usually**	**7 Every time**
11. Speaking slowly							
12. Using plain, non-medical language							
13. Show or draw pictures							
14. Limit the amount of information provided and repeat it							
15. Use the teach-back or show-me techniques *(the patients explains the information in his own words to check understanding).*							

Shared decision making	1 Never	2 Rarely	3 Occasio-nally	4 Some-times	5 Fre-quently	6 Usually	7 Every time
16. Convey awareness among patients that a choice exists in health care or treatment.							
17. Inform patients about health care or treatment options in more detail.							
18. Support patients to explore ‘what matters most to them’ after informing them on health care or treatment options.							
19. Train patients to participate in shared decision making.							
**Enabling self-management**	**1 Never**	**2 Rarely**	**3 Occasio-nally**	**4 Some-times**	**5 Fre-quently**	**6 Usually**	**7 Every time**
20. Assess barriers and facilitators relating to treatment compliance.							
21. Involve the patient in formulating personalized goals and action plans.							
22. Train patients to perform adequate self-management behaviour.							

C. Your confidence in health literacy focused communication

With respect to the following questions, please indicate your level of confidence in using your health literacy communication skills

**How confident are you in your ability to:**	**1**<br>**Not at all confident**	**2**<br>**Slightly confident**	**3**<br>**Neutral**	**4**<br>**Moderately confident**	**5**<br>**Very confident**
23. To communicate with patients who may have low health literacy					
24. Use instruments to identify patients with low health literacy					
25. Identify behaviours typically exhibited by people with low health literacy					
26. Judge appropriateness of written health information for patients with low health literacy					
27. Gather information from patients with low health literacy					
28. Provide clear information to patients with low health literacy					
29. Use the teach back or show me technique to check understanding of patients with low health literacy					
30. Create a shame free environment for patients with low health literacy					

31. Involve patients with low health literacy in shared decision making					
32. Train patients with low health literacy to participate in shared decision making					
33. Promote self-management skills in patients with low health literacy					

Do you have questions and/or comments?

Thank you very much for your cooperation!
